# Obituary

**Published:** 1886-05

**Authors:** 


### Obituary.

Sometimes it is hard to guide the pen ; and sometimes thought almost refuses to take the shape of words ; and the weight of such a time presses our pen now almost to stillness.

Jonathan Douthett, D.D.S., is dead ; and by a mysterious providence himself and wife crossed the dark river of Death, and landed on the bright shores of eternity together. Hand in hand they passed through life ; and with linked hands, and hearts that beat as one, they have passed into realms of Death.

In 1853 and 1854 Jonathan Douthett attended lectures in the medical department of the University of Michigan, not with the expectation of, nor with the intention to, practice medicine, but prepare him the better to practice dental surgery, his favorite field of professional life. The year following he attended lectures in the Ohio College of Dental Surgery, and received the degree of D.D.S. His eyes had been worsted years before by an attack of confluent small pox, and in the close study of these two years they gave way, and it became impracticable for him to practice dentistry. Hence, for some years, he carried on a fruit farm, but later he raised fine live stock, and was highly successful.

For almost forty years he has been to us as a brother beloved—a true friend, and genial companion. A member of our family,—a wife's sister—became his wife in 1858, and together they traveled life's journey, till, on the evening of April 16, 1886, their cottage home was burned to ashes, and they perished in the flames. Their pastor and other friends passed their residence between 10 and 11 P. M., and all was quiet and still, yet before midnight their bodies were burned beyond recognition.

There is no light as to the origin of the fire, and it is useless to guess at that which only eternity can reveal.

Dr. D. and wife were earnest, mature Christians, and as we recover from the fearful shock, we mourn not as those without hope, for one who has borne the burden announces himself as "the resurrection and the life."

